# Effects of Different Methionine Sources on Methionine Metabolism in the IPEC-J2 Cells

**DOI:** 10.1155/2019/5464906

**Published:** 2019-07-16

**Authors:** Fangrui Zuo, Qiongyao Gu, Shengqing Li, Hongkui Wei, Jian Peng

**Affiliations:** ^1^Department of Animal Nutrition and Feed Science, College of Animal Science and Technology, Huazhong Agricultural University, Wuhan 430070, China; ^2^Department of Chemistry, College of Science, Huazhong Agricultural University, Wuhan 430070, China; ^3^State Key Laboratory of Agricultural Microbiology, College of Science, Huazhong Agricultural University, Wuhan 430070, China; ^4^The Cooperative Innovation Center for Sustainable Pig Production, Wuhan 430070, China

## Abstract

As one of the essential amino acids, methionine (Met) plays an important role in biological events such as methylation and antioxidant properties besides its function in protein synthesis. Different Met sources have been used in animal production, but their effects on Met metabolic pathways are not well understood. In the present study, we investigated the effects of different Met sources (L-Met, DL-Met, DL-2-hydroxy-4-(methylthio)butanoic acid (DL-HMTBA), and DL-methionyl-DL-methionine (DL-MM)) on the metabolism of Met in small intestinal porcine epithelial cell line (IPEC-J2) and the contents of extracellular Met sources. The results showed that concentrations of intracellular Met, S-adenosylmethionine (SAM), S-adenosylhomocysteine (SAH), and the ratio of SAM to SAH in the DL-HMTBA group were significantly lower than that in other Met source groups, while the content of 5-methyltetrahydrofolate (5-MTHF) was significantly higher. Moreover, the mRNA levels of* MAT2A*,* AHcy*,* CBS*,* MTHFR, *and* MTR* in the DL-HMTBA group were significantly higher than those in other Met source groups. Further study showed that the total content of extracellular Met sources was highest in the DL-HMTBA group, followed by DL-MM group, followed by L-Met and DL-Met groups. These results demonstrated that DL-HMTBA mainly affects the transmethylation and remethylation of Met and it can promote the trans-sulfur metabolism of Met when compared with other Met sources. In addition, most DL-HMTBA and a small amount of DL-MM can escape the intestinal first-pass metabolism and then provide more extracellular Met sources than L-Met and DL-Met. Therefore, this study can provide a theoretical basis for the selection of Met sources in livestock.

## 1. Introduction

Methionine (Met) is the only sulfur-containing essential amino acid, which plays an important role in the normal growth of animals, such as poultry, monogastric animals, and ruminants [1–3]. Synthetic Met additives have been widely used in livestock production to supplement the deficiency of Met in natural feed ingredients. DL-methionine (DL-Met) and DL-2-hydroxy-4-(methylthio)butanoic acid (DL-HMTBA) are common Met additives. In addition, DL-methionyl-DL-methionine (DL-MM) is an efficacious source of the essential amino acid L-Met for fish and crustaceans [4]. DL-Met is a DL racemic mixture of the Met, DL-HMTBA is a DL racemic mixture of a naturally occurring organic acid [5], and DL-MM is a dipeptide formed by dehydration and condensation of two DL-Met molecules. They can all be converted to L-Met under the action of the corresponding enzyme.

Studies have shown that different Met sources are different in the intestinal first-pass metabolism. It has been found that only 48% of dietary Met intake was absorbed into the portal blood of piglets [6]. Fang et al. (2010) reported that 30% of dietary Met was metabolized by the intestine in the first pass [7], and DL-HMTBA is more likely to bypass the first-pass intestinal metabolism than DL-Met [8]. Isotope dilution infusion study indicated that the recovery of DL-HMTBA at the portal vein of lambs was 87% [9]. In addition, studies have shown that the absorption rate of Met was greater when the Met source is L-methionyl-L-methionine (L-MM) rather than L-Met [10]. Taken together, different Met sources have different metabolic degrees in the intestinal tract.

Researches showed that Met used by the gastrointestinal tissues was metabolized into homocysteine (31%), CO_2_ (40%), or tissue protein (29%) [11]. In addition to being a substrate for protein synthesis, significant transmethylation and transsulfuration of Met was demonstrated in the gastrointestinal tissues, representing about 27% and 23% of whole-body fluxes, respectively [11]. In animals, Met metabolism mainly involves the following metabolic pathways: under the catalysis of Met adenosyltransferase (MATs), Met produces S-adenosylmethionine (SAM), which can be transmethylated to S-adenosylhomocysteine (SAH) [12]; SAM is an important methyl group donor for the synthesis of creatine, lecithin, and epinephrine and also can serve as a methylation source for the methylation of DNA, RNA, and proteins [13]. The ratio of SAM to SAH is known as a methylation index [14]. Furthermore, SAM is involved in polyamine synthesis, along with the generation of 5-methylthioadenosine (MTA), which can be recycled to generate Met [15]. In the transsulfuration of Met, homocysteine (Hcy) can be further catabolized to cysteine (Cys). Hcy is an important intermediate of Met metabolism; hyperhomocysteinemia has been identified as a risk factor for a multitude of diseases [16]. Cys can become conditionally indispensable in particular situations such as stress conditions or inflammatory states [17]. In addition, Cys is a precursor for the synthesis of glutathione (GSH) and taurine, which have important antioxidant functions [18]. In the remethylation of Met, 5-methyltetrahydrofolate (5-MTHF) and betaine can provide methyl donors to Hcy to generate Met [19, 20].

It is known that the first-pass intestinal metabolism of Met and DL-HMTBA is subsistent. However, it is unclear whether DL-MM can be provided to parenteral tissues in the form of a complete dipeptide. The metabolism of Met is associated with many important physiological functions; studies have shown that different Met sources (DL-Met and DL-HMTBA) can affect the gene expression of key enzyme in Met metabolism, but whether different Met sources affect the content of corresponding metabolic pathway products is unknown. So, it is necessary to study the effects of different Met sources on the generation of Met metabolites in intestinal metabolism.

The small intestinal porcine epithelial cell line (IPEC-J2) is a nontransformed, nontumorigenic small intestinal cell line, which conserves its epithelial nature and maintains its differentiated characteristics and exhibits strong similarities to primary intestinal epithelial cells [21]. Therefore, we used IPEC-J2 cell line to study the effects of different Met sources on the contents of intracellular Met metabolites and the extracellular Met sources. The results showed that the intracellular content of Met, SAM, and SAH and the ratio of SAM to SAH in DL-HMTBA group were significantly lower than that in the other Met source groups. Most DL-HMTBA and a small amount of DL-MM can escape the intestinal first-pass metabolism and then provide more extracellular Met sources than L-Met and DL-Met. Therefore, this study is helpful for us to choose appropriate Met additives in livestock production.

## 2. Materials and Methods

### 2.1. Chemicals and Regents

Met, SAM, SAH, MTA, Hcy, Cysta, Cys, GSH, 5-MTHF, VB6, VB9, VB12, LC-MS-grade acetonitrile (ACN), LC-grade methanol (MeOH), formic acid, ascorbic acid, sodium hydroxide (NaOH) 0.1 M, hydrochloric acid (HCl), ammonium acetate (NH4OAc), tris-(2-carboxyethyl)phosphine (TCEP), L-Met, and DL-Met were purchased from Sigma-Aldrich (St. Louis, MO, USA). DL-HMTBA was purchased from Macklin (Shanghai, China). DL-MM was purchased from Taopu (Shanghai, China). Homocystine-d8 was purchased from CDN Isotopes (Pointe-Claire, Quebec, Canada). Ultrapure water was obtained from a Millipore-Q water system (Millipore, Bedford, MA, USA). Dulbecco's modified Eagle's medium and Ham's F-12 nutrient mixture (DMEM/F12), RPMI-1640 medium, fetal bovine serum (FBS), and penicillin–streptomycin were purchased from Gibco (Shanghai, China).

### 2.2. Preparation of Pig Hepatocytes

Liver cells were prepared from 7-day-old male pigs, weighing 2.6 kg. The piglet was obtained from the University's breeding farm and killed by an iv injection with pentobarbital-sodium (150 mg/kg body weight). Liver cell preparation was performed with slight modification of the 2-step collagenase perfusion technique originally described by Seglen [22]. Cells were cultured using RPMI-1640 medium without phenolic red and supplemented with 10% (v/v) foetal bovine serum, hepatocyte growth factor (10ng/ml), epidermal growth factor (20ng/ml), dexamethasone (40 *μ*g/ml), insulin (10 *μ*g/ml), and penicillin–streptomycin (100IU/ml) at 37°C in 5% CO_2_-humidified chamber. Medium were refreshed every 24 h.

### 2.3. Cell Culture and Treatment

IPEC-J2 cell line was a kind gift from Dr. He Qigai (Huazhong Agricultural University, Wuhan, China). Cells were used between passages 70 and 90. The cells were grown in the DMEM/F12 containing 10% FBS and penicillin–streptomycin (100IU/ml) at 37°C in 5% CO_2_-humidified chamber. The medium was changed every 2 days. Cells were cultured in serum-free medium for 18 h and then in Met-free medium for 6 h and finally treated with different Met source in the medium for 2 h.

### 2.4. Transepithelial Electrical Resistance Measurements

IPEC-J2 cells was seeded onto six-well Transwell system plates (Corning Inc., New York, NY, USA) and incubated for 21 d to obtain an integrated cell monolayer. The culture plate inserts consist of two chambers separated by a polycarbonate membrane (growth surface area 4.67 cm^2^; membrane pore size 0.4 *μ*m). Briefly, 3 × 10^5^ IPEC-J2 cells in 1.5 mL medium were seeded in the apical chamber that bathed in the basal chamber with 2.6 mL of medium. The changes of transepithelial electrical resistance (TEER) were measured with a Millicell-ERS instrument (Millipore, Bedford, MA, USA). Values were corrected for background resistance contributed by the insert membrane and calculated as *Ω* · cm^2^.

### 2.5. Real-Time Quantitative PCR

RNA was isolated from IPEC-J2 cells using TRIzol reagent (Life Technologies, Merelbeke, Belgium) according to the manufacturer's instructions and followed by DNase digestion using a DNA-free kit (Ambion, Foster City, CA, USA) according to the manufacturer's instructions. A total of 2 *μ*g RNA was used to prepare cDNA using oligo(dT)12-18 as a primer, the cDNA was diluted into 20 times for real-time quantitative PCR (q-PCR), and the samples were run in 10 *μ*L reaction system with SYBR GREEN qPCR mix (Bio-Rad, Richmond, CA, USA). Relative mRNA levels of genes were quantified by using a Bio-Rad CFX Connect™ Real-Time PCR Detection System (Bio-Rad, Richmond, CA, USA). Following that, gene expression levels were calculated after its normalization to the standard housekeeping gene *β*-actin using ΔΔCT method. Finally, the mean of the triplicate cycle thresholds (CT) of the target gene was normalized to the mean of triplicate CT of the reference *β*-actin using the calculation formula “2^CT^_*β*-actin_ ^−CT^_target  gene_” indicating a relative value as a fraction of the target gene. The following primers (shown in Table 1) were synthesized from Sangon (Shanghai, China).

### 2.6. Analysis of Met-Related Metabolites and Cofactors by LC-MS/MS

Cells were lysed in 1 mL TCEP solution and then incubated for 10 min at laboratory temperature and sonicated for 2 × 1 min in a pulse mode. 200 *μ*L of cell lysate was transferred to a vial containing 800 *μ*L methanol + 1% FA and vortexed for 10 s and then stored at −20°C for 2 h. The sample was centrifuged at 13000 rpm for 10 min at 4°C and the supernatants were pipetted and filtered through a 0.22-*μ*m filter and placed into vials for LC-MS/MS analysis. The LC-MS/MS analysis was performed using a LCMS-8050 triple quadrupole mass spectrometer (Shimadzu, Kyoto, Japan) equipped with an LC-30AD system (Shimadzu, Kyoto, Japan) and SIL-30AD autosampler (Shimadzu, Kyoto, Japan). Chromatographic separation was performed using gradient elution on a reversed-phase UPLC XSelect HSS T3 1.8 *μ*m, 100 × 2.1 mm I.D. column (Waters, Milford, MA, USA). The mobile phases were as follows: A, 10 mM ammonium acetate in water and B, 20% (v/v) acetonitrile in methanol. The flow rate was 0.3 mL/min. The gradient employed was as follows: 0.01–1.5 min started at 5% B, 1.5–6 min from 5% to 50% B, 6–6.1 min from 50% to 95% B and hold for 2 min, and 8–9 min from 95% to 5% B and hold for 4 min. The total time was 13 min. The sample temperature in the autosampler was maintained at 4°C, and the injection volume was 1 *μ*L in each run.

### 2.7. Statistical Analysis

All data plots were performed using GraphPad Prism 5.0 (GraphPad Software Inc., La Jolla, CA, USA). Data were presented as mean ± SEM. Differences between group means were determined by one-way ANOVA using SAS 8.0 software. It was considered statistically significant at *P* value < 0.05 and statistically extremely significant at *P* value < 0.01.

## 3. Results

### 3.1. Effects of Different Met Sources on Met Metabolites and Related Coenzymes

**Figure 1:**
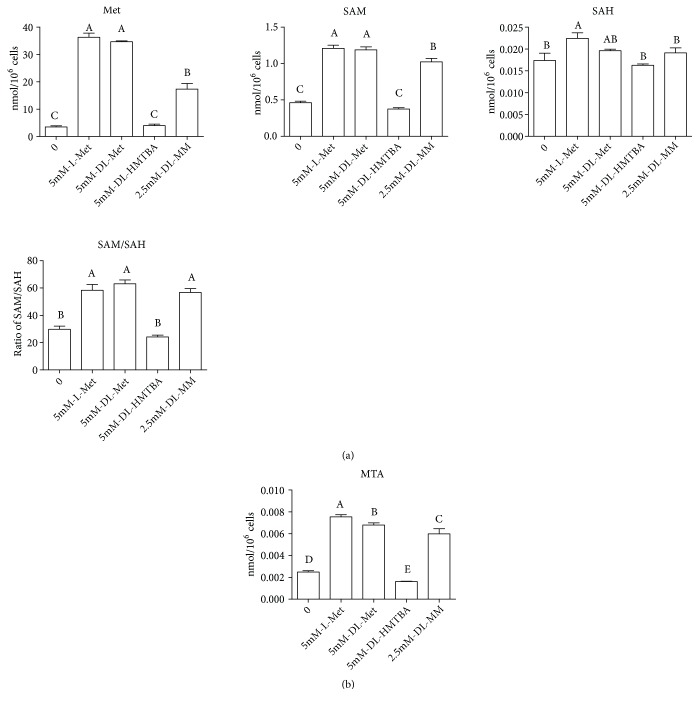
Effects of different Met sources on the contents of transmethylation-related metabolites (a) and aminopropyl transfer-related metabolite (b) in IPEC-J2 cells. Cells were seeded on cell dishes and cultured in DMEM/F-12 medium for 24 h, then cultured in serum-free medium for 18 h and then in Met-free medium for 6 h, and finally treated with different Met sources in the medium for 2 h. Shown are representative 3 independent experiments. The data are expressed as the mean ± SEM. A, B, C, D, E means without the same letter had extremely significant difference,* P* < 0.01.

Cells were incubated in medium lacking Met for 6 h and then treated with different Met sources for 2 h (the selection basis of different Met source concentrations is shown in Figure S1); at last the content of intracellular Met metabolites and related coenzymes were measured. In the process of Met transmethylation, the concentrations of intracellular Met and SAM were significantly increased in the L-Met, DL-Met, and DL-MM groups compared to those in the Met starvation group, and there was no significant difference between the Met starvation group and DL-HMTBA groups. The concentrations of intracellular SAH were significantly higher in the L-Met group than that in the other groups. There was no significant difference in the ratio of SAM to SAH among the L-Met, DL-Met, and DL-MM groups, which were significantly higher than those in the Met starvation group and DL-HMTBA group (Figure 1(a)).

In addition to the metabolism of SAM to generate SAH, a small amount of SAM undergoes the reaction of aminopropyl transfer to generate polyamines, during which MTA will be produced. Among the different Met source groups, the intracellular MTA concentration was L-Met>DL-Met>DL-MM>DL-HMTBA (Figure 1(b)).

**Figure 2:**
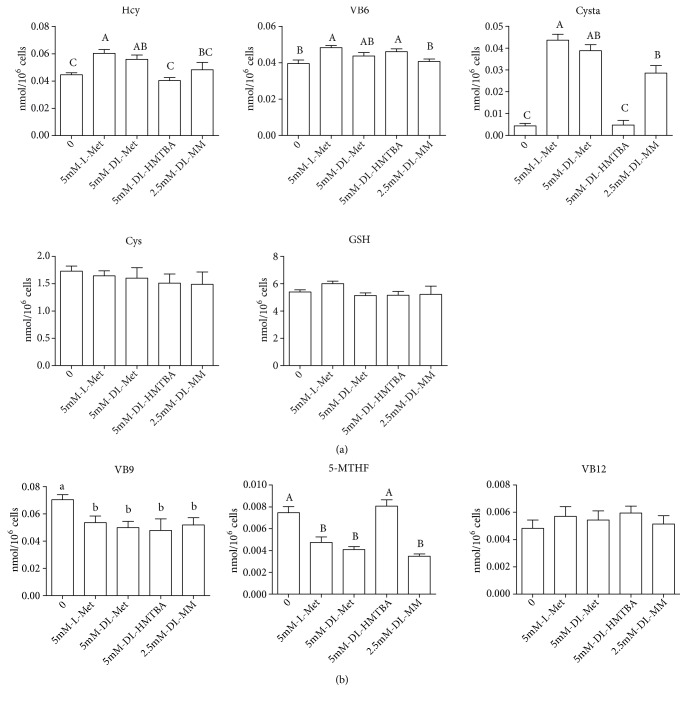
Effects of different Met sources on the contents of transsulfuration-related metabolites and cofactors (a) and remethylation-related metabolites and cofactors (b) in IPEC-J2 cells. Cells were seeded on cell dishes and cultured in DMEM/F-12 medium for 24 h, then cultured in serum-free medium for 18 h and then in Met-free medium for 6 h, and finally treated with different Met sources in the medium for 2 h. Shown are representative 3 independent experiments. The data are expressed as the mean ± SEM. A, B, C means without the same letter had extremely significant difference,* P* < 0.01; a, b means without the same letter had significant difference,* P* < 0.05.

In the process of Met transsulfuration, compared with the Met starvation and DL-HMTBA groups, the concentrations of intracellular Hcy were significantly increased in the L-Met, DL-Met, and DL-MM groups. The content of Cysta in L-Met and DL-Met group was significantly higher than that in other Met source groups. As a coenzyme for sulfur-transfer metabolism, VB6 was significantly higher in the L-Met and DL-HMTBA groups than that in other groups. There was no significant difference in the concentration of Cys among the groups, as well as GSH (Figure 2(a)).

In the process of Met remethylation, there was no significant difference in the concentration of VB12 in each group; VB9 was significantly higher in the Met starvation group than the other groups. 5-MTHF was significantly higher in the Met starvation and DL-HMTBA groups than the other groups (Figure 2(b)).

### 3.2. Effects of Different Met Sources on the mRNA Expression Levels of Key Metabolic Enzymes in Met Metabolism

**Figure 3:**
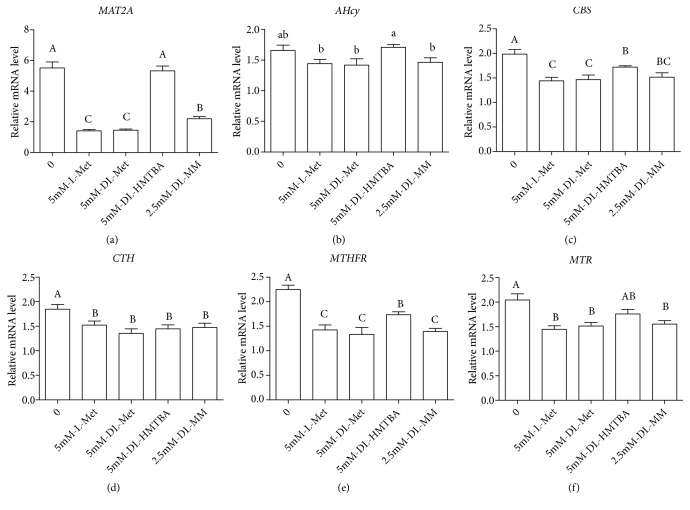
Effects of different Met sources on mRNA expression levels of the key enzymes involved in Met metabolism in IPEC-J2 cells. The mRNA expression of the Met transmethylation relative genes is shown in (a) and (b), the mRNA expression of the Met transsulfuration relative genes is shown in (c) and (d), and the mRNA expression of the Met remethylation relative genes is shown in (e) and (f). Cells were seeded onto six-well Transwell system plates and cultured in DMEM/F-12 medium for 21 d, then cultured in serum-free medium for 18 h and then in Met-free medium for 6 h, and finally treated with different Met sources in the medium for 2 h. Shown are representative 3 independent experiments. The data are expressed as the mean ± SEM. A, B, C, means without the same letter had extremely significant difference,* P* < 0.01; a, b means without the same letter had significant difference,* P* < 0.05.

Next, we investigated the mRNA levels of key enzymes involved in the Met metabolic pathway. The mRNA levels of* MAT2A* in the Met starvation and DL-HMTBA groups were significantly higher than that in other groups (Figure 3(a)). Compared with other Met sources, DL-HMTBA can significantly improve the mRNA level of* AHcy* (Figure 3(b)) and* CBS *(Figure 3(c)). There is no significant difference in the mRNA expression level of* CTH* among different Met sources (Figure 3(d)). The mRNA levels of* MTHFR* (Figure 3(e)) and* MTR* (Figure 3(f)) in the Met starvation and DL-HMTBA groups are significantly higher than that in other groups, while there was no significant difference among the other three groups.

### 3.3. Effects of Different Met Sources on the Contents of Extracellular Met Sources

**Figure 4:**
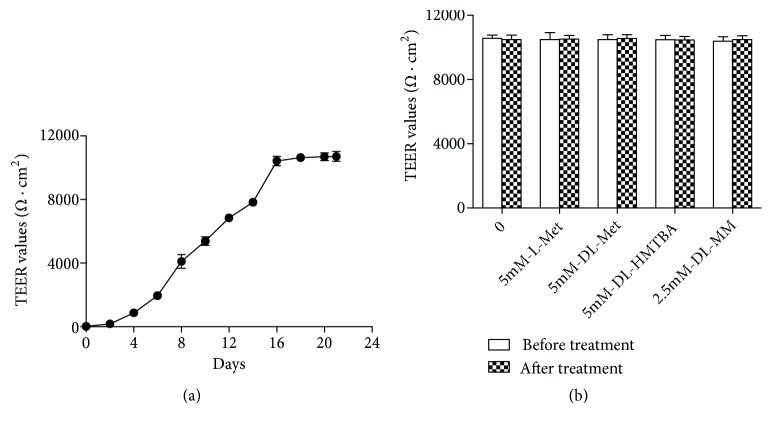
TEER values of IPEC-J2 cells cultured for 21 d (a) and the TEER values of IPEC-J2 cells after treatment (b). Cells were seeded onto six-well Transwell system plates and cultured in DMEM/F-12 medium for 21 d, then cultured in serum-free medium for 18 h and then in Met-free medium for 6 h, and finally treated with different Met sources in the medium for 2 h. Shown are representative 3 independent experiments. The data are expressed as the mean ± SEM.

**Figure 5:**
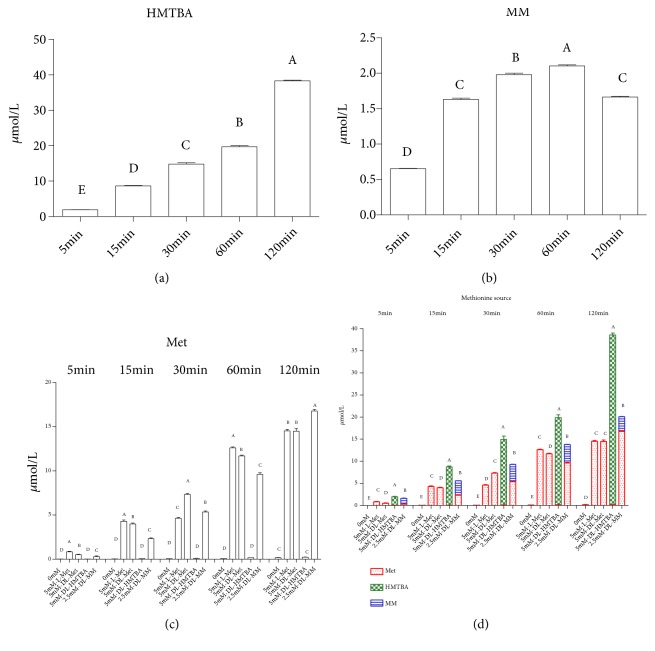
Contents of extracellular different Met sources of IPEC-J2 cells. The concentration of DL-HMTBA is shown in (a), the concentration of DL-MM is shown in (b), the concentration of Met is shown in (c), and the concentrations of total Met sources are shown in (d). Cells were seeded onto six-well Transwell system plates and cultured in DMEM/F-12 medium for 21 d, then cultured in serum-free medium for 18 h and then in Met-free medium for 6 h, and finally treated with different Met sources in the medium for 2 h. Shown are representative 3 independent experiments. The data are expressed as the mean ± SEM. A, B, C, D, E means without the same letter had extremely significant difference,* P* < 0.01.

The metabolism of different Met sources is different in IPEC-J2 cells; does it affect the contents of Met sources provided to the extracellular? Therefore, we used Transwell chamber to culture IPEC-J2 cells for 21 d and then measure the concentrations of extracellular total Met sources. It can be seen that the TEER values begin to rise significantly with the increase of time from the 4th day, while it tends to be stable during the 16 d-21 d (Figure 4(a)). The cells were cultured for 21d, then cultured in serum-free medium for 18 h and then in Met-free medium for 6 h, and finally treated with different Met source in the medium for 2 h. After the above treatment, TEER values were detected to check the integrity of cell membrane. Different Met sources had no significant effect on the TEER values of IPEC-J2 cells (Figure 4(b)). Next, the culture medium of the lower compartment was collected to detect the content of Met, DL-HMTBA, and DL-MM. It was found that the extracellular concentration of DL-HMTBA increased with time in the DL-HMTBA group (Figure 5(a)). In the DL-MM group, extracellular DL-MM increased significantly at 5, 15, 30, and 60 min but decreased significantly at 120 min (Figure 5(b)). The extracellular Met content of L-Met and DL-Met group was significantly higher than that of the other groups at 5, 15, 30, and 60 min. At 120 min, the extracellular Met content of DL-MM group was significantly higher than that of the other groups. At each time point, the extracellular Met of DL-HMTBA group was significantly lower than that of the other Met source groups (Figure 5(c)). Finally, the total extracellular Met source concentration of each treatment group was detected. The results showed that the extracellular total Met source concentration was highest in the DL-HMTBA group, followed by the DL-MM group, and lowest in the L-Met and DL-Met groups (Figure 5(d)).

### 3.4. The mRNA Levels of Key Enzymes Responsible for Converting Different Met Sources to L-Met in the Primary Hepatocytes of Pigs and IPEC-J2 Cells

**Figure 6:**
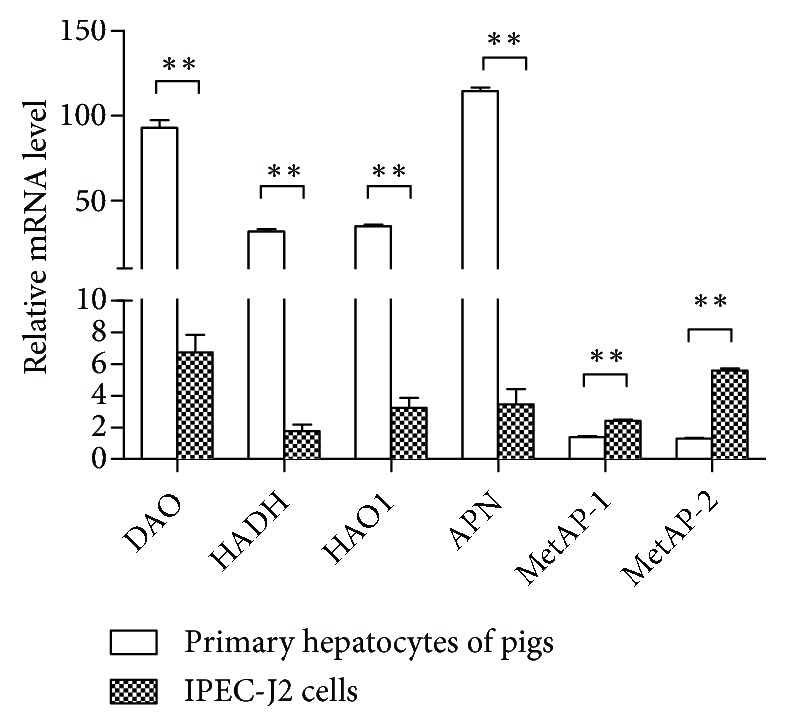
The mRNA expression levels of the key enzymes responsible for conversion of different Met sources to L-Met in IPEC-J2 cells and the primary hepatocytes of pigs. The IPEC-J2 cells and the primary hepatocytes of pigs were seeded on 6-well plates and, respectively, cultured in DMEM/F-12 medium and RPMI-1640 medium for 24 h. Shown are representative 3 independent experiments. The data are expressed as the mean ± SEM. *∗∗* means that the difference between the two groups is extremely significant,* P *< 0.01.

Aiming to investigate the possible reasons for the difference in the intracellular Met concentration in different Met sources groups, we studied the mRNA levels of key enzymes responsible for converting different Met sources to L-Met in the primary hepatocytes of pigs and IPEC-J2 cells. The mRNA levels of* DAO*,* HADH, *and* HAO1* were significantly lower in IPEC-J2 cells than that in the primary hepatocytes of pigs.* APN*,* MetAP-1, *and* MetAP-2* are involved in the hydrolysis of DL-MM. Among them, the mRNA level of* APN* was significantly lower than that in the primary hepatocytes of pigs, while* MetAP-1* and* MetAP-2* were significantly higher (Figure 6).

## 4. Discussion

Currently, different Met sources can be provided to animals during production. Studies have found that part of HMTBA and Met can be metabolized by the intestine in the first pass [6–9]. However, no study on the intestinal first-pass metabolism of DL-MM has been reported. In this study, we used the Transwell chamber to study the metabolic characteristics of Met sources such as L-Met, DL-Met, DL-HMTBA, and DL-MM in the intestine. We used the Transwell chamber to culture IPEC-J2 cells in vitro, which had been realized and positively validated, electrophysiologically as well as morphologically [23]. In this study, after intestinal metabolism, DL-MM can provide free Met and DL-MM to the extracellular tissues. At 120 min, the extracellular DL-MM decreased significantly, which may be due to the increased hydrolysis of DL-MM, resulting in a decrease in DL-MM and an increase in Met at the corresponding time point. Compared with L-Met and DL-Met, DL-HMTBA and DL-MM can provide more Met sources to the extraintestinal tissues. Studies have shown that MM can provide more free Met than L-Met [24]; DL-HMTBA can escape the first-pass metabolism of the intestine more than DL-Met [8]. It can be seen that results in this study are consistent with the above literature reports.

Different Met sources affect the contents of Met metabolites and the mRNA levels of key enzymes. The results showed that the intracellular Met concentration of DL-HMTBA group was significantly lower than that of other Met source groups. Since L-MET is the only Met directly utilized by organisms, the activities of enzymes essential for conversion of Met precursors might play an important role in determining the availability of Met [25]. The genes of rate-limiting enzymes for conversion of D-Met, D-HMTBA, and L-HMTBA to L-Met are D-amino acid oxidase 1 (*DAO1*), D-2-hydroxyacid dehydrogenase (*HADH*), and hydroxyacid oxidase 1 (*HAO1*) [26]. The N-terminal Met from oligopeptides can be split off by APN, MetAP1, and MetAP2 [27–29]. In this study, the mRNA levels of* DAO*,* HADH,* and* HAO1 *in IPEC-J2 cells are significantly lower than those in primary hepatocytes of pigs (Figure 6), which is consistent with previous research [30]. Combined with the concentration of Met in each Met source group, it can be seen that differential expression of these enzymes responsible for converting to L-Met results in different transformation degrees of Met sources in IPEC-J2 cells, thus affecting the intracellular Met concentrations.

Shiraki et al. [31] demonstrated that Met deprivation leads to a reduction in the concentration of SAM in ES and iPS cells. In our study, the contents of intracellular SAM in the Met starvation and DL-HMTBA groups were significantly lower than that in other groups, which was consistent with the trend of intracellular Met concentration. However, the expression of* MAT2A* was increased in the Met starvation and DL-HMTBA groups, which is the gene encoding for an essential cellular enzyme responsible for SAM biosynthesis [32]. The concentration of SAM has a strong negative feedback inhibition on the isoenzyme MAT2A. When the concentration of SAM increases, MAT2A is inhibited by negative feedback to maintain tissue polyamine levels and methylation [33]. The possible explanation is that, in order to maintain the content of methyl donor SAM, the body compensatory mechanism increases the expression of* MAT2A *in the Met starvation and DL-HMTBA groups. Beside, SAM is the substrate for transmethylation and aminopropyl transfer of Met. In this study, the intracellular concentrations of SAH, Hcy, and MTA and the ratio of SAM to SAH in the DL-HMTBA group were significantly lower than those in the other Met source groups and the concentration trend was consistent with the intracellular Met content, indicating that different Met sources could regulate the intracellular Met concentration and then affect the Met methylation metabolism.

In the trans-sulfur reaction of Met, the content of cysta in each treatment group showed the same trend as that of Hcy, which is the substrate of trans-sulfur reaction. However, in the DL-HMTBA group, the concentrations of Cys and GSH, which are the further metabolites of Cysta, were not significantly different from that in other Met source groups. Further studies have found that DL-HMTBA can significantly increase the mRNA level of* CBS* and there was no significant difference in the mRNA level of CTH among each group.* CBS* is the gene encoding for an enzyme responsible for catalyzing Hcy to generate Cysta and* CTH *is responsible for generating Cys from Cysta. Therefore, it is suggested that the changes in gene expression of* CBS* and* CTH *may lead to no significant difference in intracellular contents of Cys or GSH in each Met source group. This may be due to the fact that DL-HMTBA has a tendency to promote the trans-sulfur reaction compared with other Met sources to ensure the content of these important substances such as Cys and GSH. Studies have shown that, in comparison to L-Met, DL-HMTBA is preferentially diverted to the transsulfuration pathway, thus leading to a higher mucosal content of Tau in chicken small intestine [34]. When fed DL-HMTBA compared to DL-Met, higher plasmatic levels of Tau in pigs and chickens were reported [8, 35]. In addition, Martin-Venegas et al. [36] reported the protective role of DL-HMTBA in intestinal epithelia barrier function is correlated with higher taurine and the reduced form of glutathione in the Caco-2 cells, whereas DL-Met was not. Therefore, DL-HMTBA can maintain the level of antioxidants in the body by promoting the Met transsulfuration.

In the remethylation process of Met, the concentrations of 5-MTHF in the Met starvation and DL-HMTBA groups were significantly higher than that in the other groups. Correspondingly, the mRNA levels of* MTHFR* and* MTR* in the Met starvation and DL-HMTBA groups were significantly higher than those in other groups, while there was no significant difference among the other three groups. This may be due to the fact that the intracellular Met contents of these two groups were significantly lower than that of the other groups, while the difference of Met content in the other three groups was small. In order to maintain the cell demand for Met, the remethylation of Met in IPEC-J2 cells was increased.

In terms of the contents of Met metabolites and the mRNA levels of metabolic enzymes, DL-HMTBA was significantly different from L-Met, DL-Met, and DL-MM, and there was no significant difference among those three Met sources. These metabolic differences may be caused by the effects of DL-HMTBA on the expression of genes related Met conversion and metabolism. In this study, the ratio of SAM to SAH in the DL-HMTBA group was significantly lower than that of other Met sources, and the ratio of SAM to SAH is an indication of methylation capacity [14]. Changes in methylation capacity may be an important factor affecting those related genes' expression, which needs to be proved by subsequent experiments.

## 5. Conclusions

In conclusion, different Met sources are metabolized differently in the IPEC-J2 cells. Compared with L-Met, DL-Met and DL-MM have the same effect on the contents of Met metabolites and the levels of mRNA expression of metabolic enzymes. However, in the DL-HMTBA group, the contents of intracellular Met and methylation related metabolites were lower; at the same time, the ratio of SAM to SAH was significantly lower than that of the other Met source groups, while different Met sources had no significant effect on the contents of Cys and GSH, suggesting that DL-HMTBA can improve the trans-sulfur metabolism of Met to ensure the production of important antioxidants even though it did not generate Met very well. The total content of extracellular Met sources provided by different Met sources was DL-HMTBA > DL-MM > L-Met and DL-Met, respectively. Among them, DL-HMTBA mainly provided the Met sources to the parenteral tissue in the form of DL-HMTBA and DL-MM could be provided in the form of DL-MM and free Met. This study provides novel insights into selection of Met sources in livestock and poultry production.

## Figures and Tables

**Table 1:** Selected genes and primers used in this study.

Gene	Primer sequences	Product size (bp)	Tm (°C)
*β-actin*	F: CCAGGTCATCACCATCGG	158	60
	R: CCGTGTTGGCGTAGAGGT		
*MAT-2A*	F:CACTTTGCCTTGGTTACGCC	85	52
	R:TCTGATGGGAAGCACAGCAC		
*AHcy*	F: CGGACACTTTGACGTGGAGA	93	62
	R: AACAAGTAGCGGTCCACCTG		
*CBS*	F: TGCTCACTACGACATCACAGC	127	60
	R: GCACTTCTCCTTCAGCTTCCT		
*CTH*	F: GGTTCCAACATTTCGCCACG	138	61
	R: ACTCAAAACCCGAGTGCTGT		
*MTHFR*	F:AGACCATACTGCACATGACCTG	155	61
	R:GTAGCTGAAGCCTCCTTCCTC		
*MTR*	F:TTGGAGGATGCTGTGGTA	159	55
	R:TAACGAAGTTGGTGTATGGT		
*DAO*	F:GATGCCCCTTGGCTGAAGAA	173	62
	R:CAGCCTTCCCAGATGGTGTT		
*HADH*	F: GCCATCGTGGAGAACCTGAA	159	62
	R:GAAATGGAGCCCGGCAAATC		
*HAO1*	F:CGGAATGTGGCTGAAGTAGACC	136	62
	R:TCCTACAGGCTCTCACGGTTG		
*APN*	F: CAATATGCCGCCCAAAGGTTC	163	61
	R: CCGGATCAGGACGCCATTT		
*MetAP-1*	F:GATTTGTGAAGGCGGATGGC	132	63
	R:CGGGTTAGGATCTCACAGCC		
*MetAP-2*	F:CATGCTGGGAAAACAGTGCC	108	61
	R: AACAATGCCTTTGCCTGTGC		

## Data Availability

All results have been presented in the manuscript without additional data. The data used to support the findings of this study are available from the corresponding author upon request.

## Supplementary Materials

Supplementary Materials Figure S1 shows the effect of different concentrations of Met on the viability of IPEC-J2 cells. The IPEC-J2 cells were seeded on 96-well microplates (5000 cells/100 *μ*L/well) and cultured in DMEM/F-12 medium for 24 h, then in serum-free medium for 18 h, and in Met-free medium for 6 h. Finally, the cells were cultured in the Met-free medium (Met starvation group) or the medium containing different concentrations of Met (0.5, 1, 2, and 5 mM) for 24 h. At the end of the treatment, 10 *μ*L of a 5 mg/mL solution of MTT in PBS was added to each well and the plates were incubated at 37°C for an additional 4 h. Shown are representative 3 independent experiments. The data are expressed as the mean ± SEM. A, B means without the same letter had extremely significant difference,* P* < 0.01.
