# Supplementary material for: Effects of Different Methionine Sources on Methionine Metabolism in the IPEC-J2 Cells
Source: Biomed Res Int. 2019 Jul 16;2019:5464906. doi: 10.1155/2019/5464906 (PMC6662248; doi:10.1155/2019/5464906)

Supplementary Material

MTT assay

The cells were seeded on 96-well microplates (5000 cells/100 μL/well) and cultured in DMEM/F-12 medium for 24 h, then in serum-free medium for 18 h and in Met-free medium for 6 h. Finally, the cells were cultured in the Met-free medium (Met starvation group) or the medium containing different concentrations of Met (0.5, 1, 2 and 5 mM) for 24 h. At the end of the treatment, 10 μL of a 5 mg/mL solution of MTT in PBS was added to each well and the plates were incubated at 37°C for an additional 4 h. The supernatant was then discarded, and the purple formazan crystals formed were solubilized with 100 μL/well of DMSO (Sigma-Aldrich, St. Louis, MO, USA). The absorbance of the 96-well plate was determined using an automatic microplate spectrophotometer at 570 nm. Cell viability was calculated as the percent ratio of absorbance of the samples against the control medium.

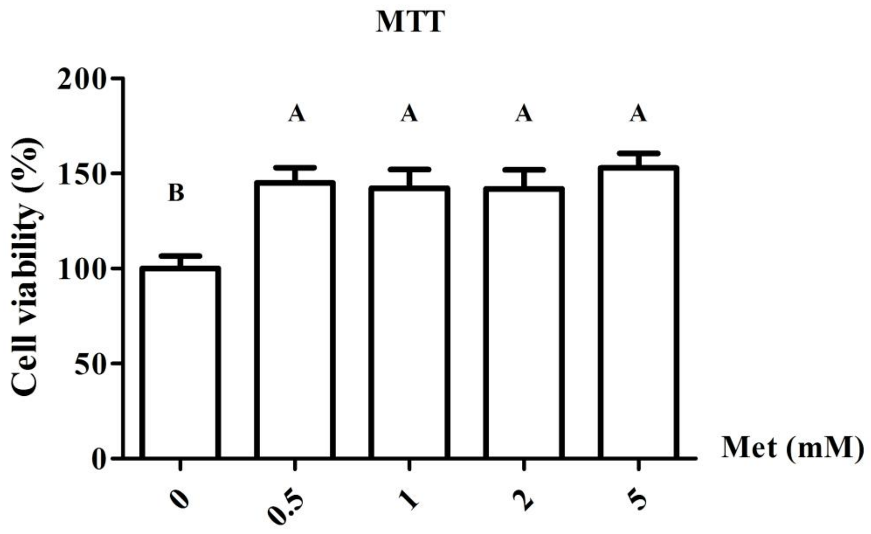

Supplement: Supplementary Materials — Figure S1 shows the effect of different concentrations of Met on the viability of IPEC-J2 cells. The IPEC-J2 cells were seeded on 96-well microplates (5000 cells/100 μL/well) and cultured in DMEM/F-12 medium for 24 h, then in serum-free medium for 18 h, and in Met-free medium for 6 h. Finally, the cells were cultured in the Met-free medium (Met starvation group) or the medium containing different concentrations of Met (0.5, 1, 2, and 5 mM) for 24 h. At the end of the treatment, 10 μL of a 5 mg/mL solution of MTT in PBS was added to each well and the plates were incubated at 37°C for an additional 4 h. Shown are representative 3 independent experiments. The data are expressed as the mean ± SEM. A, B means without the same letter had extremely significant difference, P < 0.01. [file 5464906.f1.pdf]
